# Seizures and Epilepsy: An Overview for UK Medical Students

**DOI:** 10.7759/cureus.70654

**Published:** 2024-10-01

**Authors:** Abdullah Ahmed, Abdul-Habib Rahman, Shimul Williams, Mubeen Toufiq, Eyad Jamileh, Sheema Chaudhry, Khadijah Ahmed, Munir Ahmed

**Affiliations:** 1 Family Medicine, Whipps Cross Hospital, London, GBR; 2 Cardiology, Luton and Dunstable Hospital, Luton, GBR; 3 Pathology, Princess Alexandra Hospital, London, GBR; 4 Respiratory Medicine, Princess Alexandra Hospital, London, GBR; 5 Gastroenterology, Royal Blackburn Hospital, Blackburn, GBR; 6 Critical Care Medicine, Princess Alexandra Hospital, London, GBR; 7 Gastroenterology, University of Leeds, Leeds, GBR; 8 Physics, Leyton Sixth Form College, London, GBR

**Keywords:** abnormal electrical discharges, anti-epileptic drugs, epilepsy, neurological disorders, seizures

## Abstract

Epilepsy is a common neurological disorder impacting millions globally, marked by recurrent, unprovoked seizures. This review article, tailored for UK medical students, provides a broad clinical overview of epilepsy, focusing on its pathophysiology, classification, and management strategies. The article clarifies the distinction between epilepsy and seizures and delves into key areas, including risk factors, clinical features, and differential diagnosis. The discussion extends to diagnostic methods, underscoring the importance of conducting a thorough evaluation in diagnosing and managing epilepsy effectively. Data were drawn from the UK National Institute for Health and Care Excellence (NICE) guidelines, World Health Organization (WHO) reports, and key peer-reviewed studies. Particular attention was given to UK-specific data on epilepsy incidence, treatment gaps, and clinical outcomes. A review of relevant literature was conducted, covering epidemiology, pathophysiology, diagnostic protocols, and management strategies based on UK practices. Finally, the article addresses the acute management of seizures and the pharmacological and non-pharmacological management of epilepsy based on the NICE guidelines. The goal is to offer medical students a concise yet comprehensive understanding of epilepsy, preparing them for practical, evidence-based decision-making in clinical practice.

## Introduction and background

The discovery of epilepsy can be dated back to around 2,000 B.C. in Mesopotamia, where one of the first written descriptions of epilepsy was recorded. The perception and stigmata of epilepsy have changed constantly throughout history, including in ancient Greece, where epilepsy was considered a divine punishment by the gods; in Sparta, all babies with epilepsy were left to die on Taygetus mountain [1]. The word ‘epilepsy’ is originally derived from the ancient Greek word epilamanein, which means ‘to be possessed’ or ‘to be seized’ [2]. It was not until 1875 that Hughlings Jackson, the father of epilepsy, recognised that seizures were linked to abnormal electrical discharges occurring in the brain [3].

Historically, the terms epilepsy and seizure have been used interchangeably; however, they do not refer to the same phenomenon. A seizure is an uncontrollable involuntary motor, sensory, or autonomic event caused by abnormally sustained hyper-synchronised electrical discharges occurring in the brain’s neuronal networks [4]. Seizures have many causes, one of which is epilepsy. Epilepsy is a chronic neurological disorder that manifests as recurrent, unprovoked paroxysmal seizures. The World Health Organization (WHO) defines epilepsy as a condition where the affected person has two or more unprovoked seizures occurring more than 24 hours apart [5]. Epilepsy can be considered a disease, with a seizure being a clinical manifestation or symptom of the disease.

## Review

Causes of epilepsy and seizures

There are many causes of seizures beyond epilepsy. Seizures can be classified as unprovoked or provoked. Provoked seizures are also known as acute symptomatic seizures and are essentially caused by structural or metabolic damage to the brain that alters the excitability and synchronisation of neuronal networks. When a seizure has no known cause, it is termed an unprovoked seizure. Thus, epilepsy ultimately manifests as recurrent, unprovoked seizures. There are many wide-ranging causes of provoked seizures, such as the abrupt withdrawal of alcohol from a chronic alcohol consumer, low blood glucose level, or overdosing on drugs such as cocaine, insulin, or antidepressants. Some of the common causes of provoked seizures are outlined in Table 1.

**Table 1:** Common causes of provoked seizures Table 1: common causes of provoked seizures [6]

Common causes of provoked seizures:
Withdrawal syndromes: alcohol, benzodiazepines
Metabolic: hyponatraemia, hypernatraemia, hypoglycaemia, hypocalcaemia, dehydration, high urea levels
Central nervous system infections: meningitis and encephalitis, sepsis
Space occupying lesions: tumour, abscess
Fever (especially in young children), heat intolerance
Sleep deprivation
Stroke
Head injury
Poisoning, drug abuse (cocaine, insulin, antidepressants)
Malignant hypertension
Eclampsia
Structural: cavernous malformation, arteriovenous malformation

Some of the causes outlined in Table 1 are benign, such as fever in a child; some are preventable, such as abrupt alcohol withdrawal; some are self-induced, such as overdosing on cocaine; and some are deadly, such as a brain tumour that compresses vital structures in the brain. Therefore, clinicians must always consider the numerous potential causes of provoked seizures in their differential diagnoses.

Epidemiology of epilepsy

According to the WHO, epilepsy affects approximately 50 million people globally, with 80% of cases occurring in lower- and middle-income countries [5]. According to Epilepsy Research UK, more than 600,000 people in the UK are currently living with epilepsy, with 600 new diagnoses/cases being added to the list weekly [7]. Furthermore, it is well documented that around 30% of individuals with epilepsy do not achieve adequate seizure control using the currently available antiepileptic drugs (AEDs) [7]. Moreover, only 52% of individuals with epilepsy in the UK are seizure-free, in contrast to the expected 70% [8]. This discrepancy may be attributed to incorrect diagnoses or a lack of access to optimal treatments.

To compound the problem, these pharmacoresistant patients suffer from a significantly reduced quality of life, with adverse effects on their mental health, employability, ability to drive, and marriage status. One study revealed that, in 2016, epilepsy was responsible for the loss of over 13 million disability-adjusted life years globally [9]. The resultant psychological stress should not be overlooked, as numerous studies have demonstrated that illness-related stigma can cause more suffering than the illness itself [10].

Patients with epilepsy also experience higher mortality and morbidity rates compared with the general population. The probability of these patients dying from various causes is as high as three times the average risk of the population [5]. The most common causes of death directly linked to epilepsy are sudden unexpected death in epilepsy (SUDEP), status epilepticus, unintentional injuries, and suicide [11].

Epilepsy imposes both direct and indirect costs on health services and the broader economy. Diagnosis and treatment from primary and secondary healthcare services are the leading direct costs encountered by healthcare and social services, and unemployment and premature deaths constitute the main indirect costs affecting the overall economy. A recent economic study reported that idiopathic epilepsy alone costs the UK economy £1.7 billion annually [12].

Understanding the demographics of epilepsy is essential. The difference in the incidence rate of epilepsy between men and women is minimal, but men have a slightly higher rate [13]. One systemic review concluded that the incidence of epilepsy was 50.7 per 100,000 per year for men and 46.2 per 100,000 per year for women [14]. However, people from low socioeconomic backgrounds have an increased risk of developing epilepsy, and there is a similar incidence of epilepsy across all races [15]. To conclude, socioeconomic status is a significant factor in determining the incidence of epilepsy, but not race or sex.

Classification of epilepsy

The invention of the electroencephalogram (EEG) in the 1920s, combined with advancements in neuroimaging, clinical biochemistry, and molecular genetics over the last 50 years, has dramatically improved our understanding and treatment of epilepsy [16]. However, considerable gaps in our knowledge of the aetiology and pathophysiology of epilepsy remain. The classification of epilepsy is constantly being updated in line with major scientific developments. Here, we discuss the 2017 classification of epilepsy from the International League Against Epilepsy (ILAE). In 2017, the ILAE proposed three levels for classifying epilepsy [16]:

The first level of classification (Level 1) classifies epilepsy by seizure type. This can be achieved by obtaining a thorough history of the symptoms experienced by the patient. Witnessing the seizure (for example, by video recording) or obtaining a witness account can help in classifying the seizure type. There are three main types of seizures:

Focal-onset seizures arise from only one hemisphere of the brain. This subset can be further divided into aware or impaired awareness seizures according to whether the patient loses consciousness during the seizure episode. Furthermore, the level of body movement determines whether the seizure is motor or non-motor type. If there is no body movement or limb jerking, then it is classified as non-motor.

Generalised-onset seizures originate from both hemispheres of the brain simultaneously. They can be split into motor or non-motor types based on body movement. All patients lose consciousness during a generalised seizure.

Unknown-onset seizures occur when the clinician is unsure whether the patient has a generalised or focal seizure. These can also be divided into motor or non-motor types.

**Figure 1:**
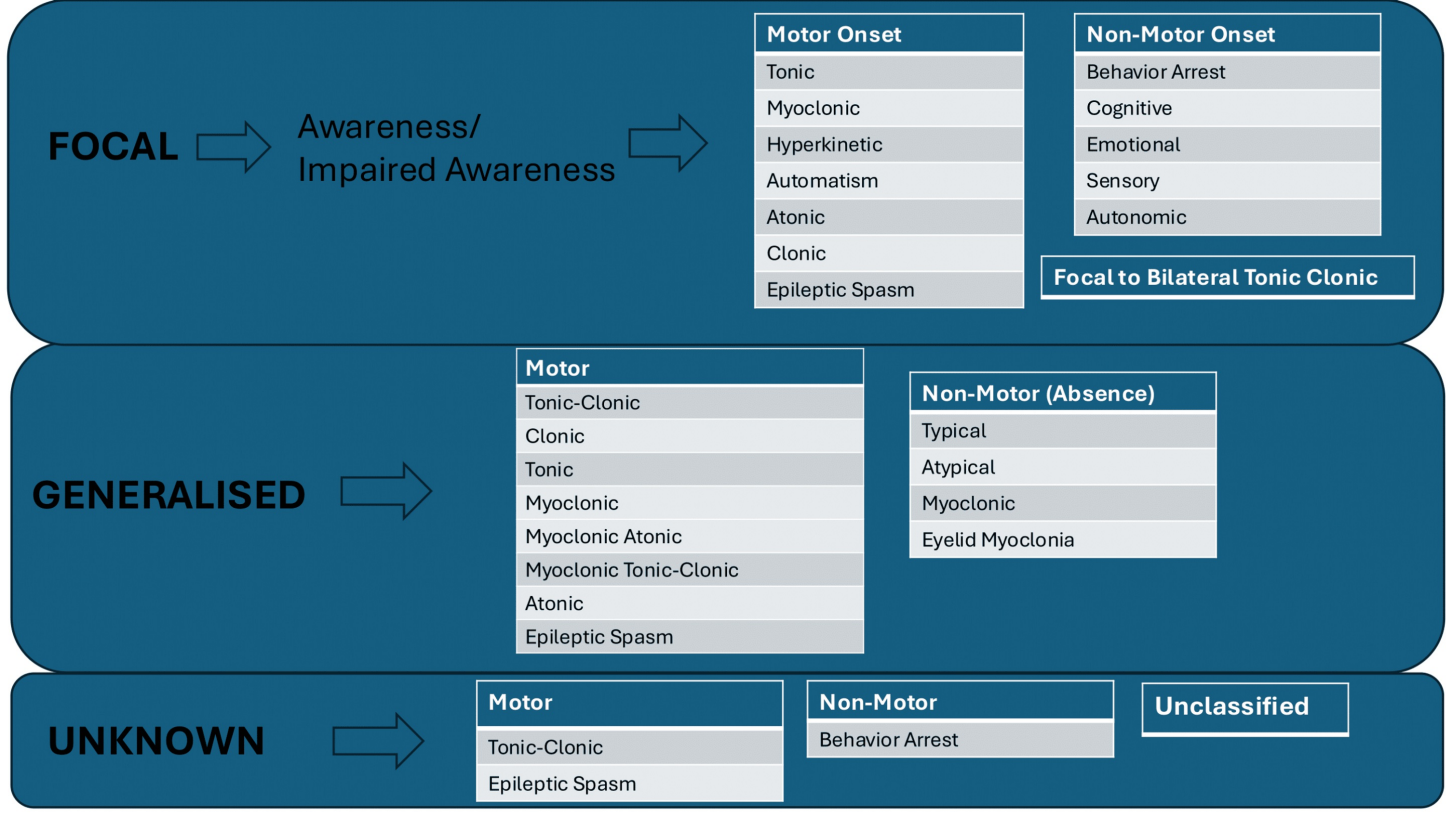
Seizure types Adapted from [17]

Figure 1 provides a summary of seizure subtypes.

The second level of classification (Level 2) classifies epilepsy by epilepsy type [18]:

Focal: In focal epilepsy, the EEG reveals focal epileptiform discharges. Key subtypes include focal motor, focal non-motor, focal aware, focal impaired awareness, and focal to bilateral tonic-clonic seizures.

Generalised: In generalised epilepsy, the EEG reveals generalised spike-wave activity. There are also subtypes of generalised epilepsy similar to those of the generalised-onset seizure types.

Combined focal and generalised: Combined focal and generalised epilepsy occurs when patients have both focal and generalised seizures. This can be observed in patients with epilepsy syndromes such as Dravet syndrome.

Unknown: Unknown epilepsy is where the patient has epilepsy, but the clinician is unsure of its type. This may result from a normal EEG reading or lack of access to an EEG (which is frequent in underdeveloped countries).

The third level of classification (Level 3) is to diagnose the epilepsy syndrome [18]. There are currently no classification systems for epilepsy syndromes provided by the ILAE. However, many epilepsy syndromes exist, with each type of syndrome having similar EEG readings, clinical features, age onset, and prognosis.

Pathophysiology of epilepsy and seizures

The pathophysiology of epilepsy is a crucial topic to explore, as it allows for a better understanding of the process of seizure/epilepsy causation. Understanding this can aid in the development and design of better-targeted AEDs. It is important to note that our current understanding of epilepsy and seizure pathophysiology is incomplete, with numerous theories available in the literature. In this section, we analyse the leading theory on seizure pathophysiology.

The first recorded scientific approach to explain epilepsy was developed by the father of medicine, Hippocrates of Kos. He disputed the mystical beliefs related to epilepsy and claimed, ‘This disease is, in my opinion, no more divine than any other; it has the same nature as other illnesses…’ [1]. Different theories of the pathophysiology of epilepsy have been proposed throughout history, such as the overproduction of black bile, which was suggested by Aristotle [19]. It was during the Renaissance period that Charles Le Pois proposed the idea that epilepsy originated from the brain, and he identified anatomical abnormalities in the brains of epileptics with epilepsy during post-mortem examinations [19]. Hughlings Jackson further supported the theory of epilepsy originating from the brain, explaining that seizures were ‘occasional, sudden, excessive, rapid, and local discharges of grey matter’ [20]. Analysing multiple EEG patterns, Gibbs and Lennox described epilepsy/seizures as ‘paroxysmal cerebral dysrhythmia’ [21].

Although the pathophysiology of seizures is not yet fully understood, the theory describing an atypical balance between excitatory and inhibitory mechanisms is well established. This theory suggests that an excess of excitatory neurotransmitters, such as glutamate, or a reduction in inhibitory neurotransmitters, such as gamma-aminobutyric acid, produces a hyper-excitable state, triggering a paroxysmal depolarising shift, causing a seizure [6]. This theory is supported by evidence that promoting the mechanisms that facilitate neuronal excitation leads to a seizure, inhibiting the inhibition mechanisms of neuronal firing causes seizures, inhibiting the promoting mechanisms prevents seizures from occurring, and promoting the inhibition mechanism also prevents seizures from occurring [22].

Many factors contribute to the abnormal excitatory and inhibitory imbalance. These include genetic factors, developmental factors, disorders of neural migration, and acquired factors. These factors can act on multiple levels in the nervous system, such as on ion channels, cell receptors, synapses, and neuronal networks [22]. Genes are directly linked to the causation and pathophysiology of some types of epilepsy syndromes, including generalised epilepsy with febrile seizures plus benign familial neonatal convulsions, autosomal dominant nocturnal frontal lobe epilepsy, and episodic ataxia type 1 with partial seizures [23].

Clinical features and diagnosis of epilepsy

This section uses information from the National Institute for Health and Care Excellence (NICE) guidelines for the diagnosis of epilepsy. There are various steps to diagnosing epilepsy. The cornerstone of the diagnosis is the clinical history obtained from the patient and/or witnesses. Additionally, laboratory investigations and neurological and cardiac examinations can help identify the cause of a provoked seizure and detect the presence of epilepsy syndromes. The use of EEG along with imaging such as magnetic resonance imaging (MRI), computed tomography (CT), single photon emission computed tomography (SPECT), and positron emission tomography (PET) plays an adjunct role in supporting the diagnosis of epilepsy and can also help classify the type of seizure and locate the epileptiform lesion [24]. The diagnosis of epilepsy can be split into five steps:

Step 1: Obtaining a history from the patient and/or witnesses

When obtaining the history from the patient or witnesses, there are three key parameters to consider:

What happened before the attack? This includes identifying any potential epilepsy/seizure triggers, including but not limited to alcohol intake, sleep deprivation, and flashing lights. Additionally, it is essential to ask whether the patient experienced an aura at the beginning of the attack and identify risk factors [25]. A list of common risk factors is provided in Table 2.

**Table 2:** Risk factors of seizures [26]

Risk Factors
Premature birth
Neurodegenerative disorders
Cerebrovascular disease
Head trauma
Infections- meningitis/encephalitis
Tumour
Family history
Brain development malformations
Genetic conditions- Tuberous sclerosis, neurofibromatosis

As shown in Table 2, the risk factors of seizures primarily involve attacks on or disorders of the body’s nervous system. This includes trauma to the head; infectious causes, such as encephalitis; and structural causes, such as a brain tumour.

The features and details of the attack itself must be determined. This includes the duration and symptoms of the attack, such as urinary incontinence, tongue biting, loss of consciousness, behavioural arrest, muscle stiffening, or rhythmic jerking of the limbs [27]. Table 3 provides a basic summary of the different symptoms experienced in specific seizures.

**Table 3:** Symptoms of epilepsy [27]

Type of Epilepsy	Symptoms
Generalised tonic-clonic	Loss of consciousness with collapse; tonic phase: generalised body stiffening; clonic phase: violent jerking; post-ictal state: deep sleep; other symptoms: tongue biting and urinary incontinence
Absence	Loss of consciousness for a few seconds, staring blankly, subjective loss of time
Myoclonic	Brief sporadic electrical shock-like jerking movements
Clonic	Violent rhythmic jerking movements
Tonic	Rigidity and stiffening
Atonic	Loss of muscle tone leading to a fall
Focal aware seizure	Awareness maintained; four subcategories: motor – jerking, stiffening and others; sensory – the five senses affected (if the patient experiences sensory symptoms only, the term aura is used); autonomic; psychological – memory, emotions or other phenomena
Focal impaired awareness seizure	Awareness impaired, automatisms

What happened after the attack? The period after a seizure is also known as the post-ictal period. During this period, the patient experiences symptoms such as headaches, fatigue, and amnesia, which further support the diagnosis of a seizure. The post-ictal phase can last from a few minutes to several hours, depending on the individual and the type of seizure experienced.

It is important to remember that the diagnosis of epilepsy requires at least two unprovoked seizures more than 24 hours apart.

Step 2: Performing a physical examination

A physical examination can also support the diagnosis of epilepsy, especially for epilepsy syndromes. For example, in tuberous sclerosis and neurofibromatosis, abnormal skin markings would be present [4]. The NICE guidelines recommend cardiac, neurological, and mental state examinations as well as the examination of the oral mucosa to identify tongue biting, which can occur during a seizure [28].

Step 3: Ordering laboratory investigations

If the diagnosis of a seizure is likely, it must be determined whether the seizure was provoked or unprovoked [29]. A number of investigative techniques can be used to determine the cause of a provoked seizure. A 12-lead electrocardiogram is performed to exclude causes such as cardiac arrhythmias and convulsive arrhythmias. Other laboratory investigations, such as obtaining a full blood count, urea and electrolyte levels, liver function tests, and glucose and calcium levels, are important to help exclude other differentials. The combination of history taking, physical examination, and laboratory investigations can help exclude other causes that can present similarly to epilepsy. Table 4 provides a list of some of the epilepsy differentials.

**Table 4:** Differential diagnosis of seizures/epilepsy [28]

Differential Diagnosis
Cardiac Causes: convulsive syncope, cardiac arrhythmias,
Panic attacks and hyperventilation
Psychogenic non-epileptic seizures: this is the most common misdiagnosis of epilepsy
Transient ischaemic attacks
Alcohol intoxication
Hypoglycaemia and other metabolic disorders
Delirium/dementia
Others: Vasovagal syncope

Many of the differential diagnoses mentioned in Table 4 include loss of consciousness, which is a common symptom of generalised seizures. Therefore, any patient presenting with loss of consciousness should be evaluated for these differentials. The differentials of epilepsy should always be considered when evaluating a patient for the diagnosis of epilepsy. One population-based study found that the misdiagnosis rate of epilepsy may be as high as 23% [30].

Step 4: EEG and imaging

The use of an EEG can help support the diagnosis of epilepsy by detecting abnormal electrical activity in the brain. It can also help determine whether the epilepsy is a generalised epilepsy with diffuse bilateral spikes or a focal epilepsy with focal spikes [4]. However, certain types of epilepsy, such as focal-aware epilepsy, can have negative EEG findings [24]. Using a video EEG can increase the probability of diagnosing epilepsy and help exclude other epilepsy differentials [4]. According to NICE, MRI is the imaging investigation of choice for epilepsy [29]. This method can help identify the location of a possible epileptogenic lesion and is good at detecting dysgenesis, cortical malformation, and hippocampal sclerosis [4]. Furthermore, SPECT and PET imaging can further assist in the localisation of the area responsible for seizure initiation. However, a CT scan is used only for urgent investigation or if MRI is contraindicated, as it has a low detection rate for focal lesions (30%) but is good at detecting haemorrhage, calcification, and tumours [4,24].

Step 5: Diagnostic uncertainty

If it is not possible to establish the diagnosis of epilepsy, referral to tertiary epilepsy specialists is recommended by NICE [29].

Management of epilepsy and seizures

The treatment of epilepsy has evolved throughout history, from the use of herbs and chemicals to the discovery of the first anticonvulsant potassium bromide by Sir Locock in 1857 [31]. Currently, there are multiple treatment options for managing epilepsy and acute seizures. It is important to note the management of an acute seizure differs from the management of epilepsy.

Acute seizures lasting beyond 5 minutes (known as status epilepticus) are managed with benzodiazepines followed by AEDs and, finally, general anaesthesia [32].

Management of an acute seizure: If a patient starts seizing, administer oxygen, control airways, and monitor vital signs immediately. It is important to check the patient’s blood glucose to rule out hypoglycaemia. If the patient’s seizure has continued for more than 5 minutes, then the patient has reached status epilepticus, which constitutes a medical emergency. Table 5 provides a summary of the treatment of status epilepticus.

**Table 5:** Treatment of status epilepticus [32]

Stage	Treatment	Timing
Generalised convulsive status epilepticus	Resuscitation and benzodiazepine (buccal midazolam or rectal diazepam in community, intravenous lorazepam if in hospital with intravenous access), address underlying causes (hypoglycaemia, alcohol withdrawal, eclampsia)	Start treatment 5 minutes after seizure onset
If first dose of benzodiazepine doesn't work	Administer second dose of benzodiazepine if seizure continues	Within 5-10 minutes of first dose
If two doses of benzodiazepine don't work	Intravenous second-line treatment: levetiracetam, phenytoin, sodium valproate	Seek expert advise
If second-line treatment doesn't work	Consider alternative second-line treatment option under expert guidance	Seek expert advise
If all second-line options fail	Third-line options: phenobarbital or general anesthesia	Seek expert advise

Epilepsy is managed with pharmacological drugs known as AEDs. There are well over 20 different types of AEDs available, with varying efficacies and mechanisms of action. If seizure freedom is still not achieved after AED use, other management options, such as ketogenic diet, surgery, vagus nerve stimulation, deep brain stimulation, and cannabis oil, can be tried. The initiation of treatment usually begins after the occurrence of the second seizure, unless the patient deems that a second seizure is unacceptable or there is a high chance of further seizure occurrence [29]. We will now explore the mechanism of action of AEDs and treatment based on seizure type.

Mechanism of action

The goal of AEDs is to decrease the activation of neuronal cells, thus decreasing the activation threshold of seizure occurrence. These drugs have different mechanisms of action. Table 6 provides a basic summary of the action sites of 18 different AEDs.

**Table 6:** Action site of AED's Table 6: Action site of AED's [33]

AED	Major action site	Minor action sites
Phenobarbital	GABA	-
Phenytoin	Na+	GABA
Carbamazepine	Na+	-
Ethosuximide	Ca2+	-
Sodium Valproate	Na+	GABA & Ca2+
Clobazam	GABA	-
Clonazepam	GABA	-
Vigabatrin	GABA	-
Lamotrigine	Na+	GABA & Ca2+
Oxycarbazepine	Na+	
Gabapentin	Ca2+	GABA
Felbamate	-	-
Topiramate	Na+	GABA &AMPA-R
Zonisamide	-	Ca2+ & Na+
Levetiracetam	SV2A	Ca2+
Rufinamide	Na+	-
Lacosamide	Na+	CMRP2
Perampanel	AMPA-R	-

As shown in Table 6, most AEDs have multiple receptor targets and modes of action. However, each AED has a chief action site. These drugs decrease the activation of neuronal cells through mechanisms such as blocking sodium channels (preventing the depolarisation of neurons), blocking calcium channels (preventing the release of neurotransmitters), enhancing potassium channels (hyperpolarising neurons), inhibiting glutamate excitation (discussed in the pathophysiology of epilepsy and seizures section), and promoting GABA inhibition (discussed in the pathophysiology of epilepsy and seizures section).

Treatment based on seizure type

The NICE guidelines advocate the use of the British National Formulary (BNF) in the management of epilepsy. Table 7 summarises the BNF and NICE recommendations for AED use according to seizure type [32].

**Table 7:** Choice of AED according to seizure type [32]

Seizure Type	First-line Monotherapy	Second-line Monotherapy	First-line Add-on	Second-line Add-on
Generalized Tonic-Clonic	Sodium valproate (boys, girls under 10, women unable to have children); Lamotrigine or levetiracetam (women and girls able to have children)	Lamotrigine or levetiracetam	Clobazam, lamotrigine, levetiracetam, perampanel, sodium valproate (except women and girls able to have children), topiramate	Brivaracetam, lacosamide, phenobarbital, primidone, zonisamide
Focal Seizures	Lamotrigine or levetiracetam	Carbamazepine, oxcarbazepine, zonisamide	Carbamazepine, lacosamide, lamotrigine, levetiracetam, oxcarbazepine, topiramate, zonisamide	Brivaracetam, cenobamate, eslicarbazepine acetate, perampanel, pregabalin, sodium valproate (except women and girls able to have children)
Absence Seizures	Ethosuximide	Sodium valproate (boys, girls under 10, women unable to have children); Lamotrigine or levetiracetam (women and girls able to have children)	Lamotrigine or levetiracetam	Ethosuximide
Myoclonic Seizures	Sodium valproate (boys, girls under 10, women unable to have children); Levetiracetam (women and girls able to have children)	Levetiracetam	Brivaracetam, clobazam, clonazepam, lamotrigine, phenobarbital, piracetam, topiramate, zonisamide	N/A
Tonic or Atonic Seizures	Sodium valproate (boys, girls under 10, women unable to have children); Lamotrigine (women and girls able to have children)	Lamotrigine	Clobazam, rufinamide, topiramate	N/A
Idiopathic Generalized Epilepsies	Sodium valproate (boys, girls under 10, women unable to have children); Lamotrigine or levetiracetam (women and girls able to have children)	Lamotrigine or levetiracetam	Perampanel or topiramate	N/A

To summarise, after considering the different factors in AED choice, the BNF recommends the following:

Start with AED monotherapy (start with the lowest possible dose and titrate up to effective seizure control).

If Step 1 fails, begin monotherapy with a second drug (slowly withdraw the first drug while introducing the second drug).

If Step 2 fails, polytherapy may be used. This has the potential for many side effects, and drug-drug interactions may occur.

Non-pharmacological treatment

As previously noted, only around 70% of patients achieve full seizure control. Studies have shown that 63%-79% of patients achieve 1 year of seizure freedom after trialling two or three AEDs [34]. Therefore, these patients need to be managed in other ways besides pharmacological therapy. These may include surgery or a ketogenic diet.

Surgical resection: this can be highly successful, even leading to complete freedom from seizures, particularly in focal-onset epilepsy. First, MRI, SPECT, PET, and EEG are used to locate the brain tissue containing the epileptogenic focus, followed by craniotomy and resection of the tissue. It should be noted that the brain tissue resected should not be associated with the eloquent parts of the brain, as it may lead to prominent neurological deficits. Furthermore, lesionectomy can be used to remove tumour and vascular deformities that are known to be a cause of epilepsy generation [24]. If a patient does not have an identifiable epileptogenic focus, such as patients with generalised-onset and atonic epilepsy, disconnection procedures such as corpus callosotomy can be performed [34]. Although disconnection procedures are not very effective, they do allow approximately 20% of patients to achieve seizure freedom [24].

Ablative procedures: resecting epileptogenic brain tissue can be done via a procedure called radiofrequency ablation. This procedure has been assessed in mesial temporal lobe epilepsy, producing various results. The main advantages of this procedure are accurate target precision, resulting in less damage to healthy brain tissue, and the fact that it is less invasive (no craniotomy). The main disadvantage is that the benefits of the procedure only take effect after 12 months [34].

Therapeutic devices: vagus nerve and deep brain stimulation can be used in patients who are unable to undergo surgical resection procedures. Studies have shown that vagus nerve stimulation can reduce seizure frequency by 50% after 3 years of treatment [35]. Common side effects associated with vagus nerve stimulation are cough, hoarseness, and difficulty speaking.

Ketogenic diet: a ketogenic diet is a non-pharmacological and non-surgical treatment of epilepsy. In 1911, Gulpae and Marie were the first physicians to introduce the ketogenic diet as a treatment for epilepsy [31]. Essentially, a ketogenic diet is a diet consisting of high fat, low carbohydrate, and adequate protein content. This results in the body using fats as a source of energy. These fats are converted into ketone bodies, which produce their antiepileptic effects in the brain by increasing gamma-aminobutyric acid levels and converting glutamate into gamma-aminobutyric acid [24]. Studies have shown that a ketogenic diet can decrease the frequency of seizures in 30%-40% of children and is effective in patients with infantile epileptic seizure therapy [36]. Overall, a ketogenic diet is used in patients who are resistant to pharmacological treatment and unable to undergo surgery for various reasons.

Discussion

For UK medical students, understanding epilepsy's clinical and societal impact is crucial. Epilepsy affects over 600,000 individuals in the UK, with many not achieving adequate seizure control, despite adherence to NICE-approved AEDs [7]. This unmet need in epilepsy management represents a significant challenge in UK healthcare, requiring clinicians to stay abreast of both pharmacological and non-pharmacological advancements. The limitations in the current classification and treatment options also have educational significance, as they highlight the need for future UK doctors to become familiar with emerging therapies. Socioeconomic disparities are evident in UK epilepsy cases, as individuals from lower-income backgrounds experience worse health outcomes, emphasising the importance for students to recognise these factors in patient care and public health interventions.

Moreover, medical students in the UK must be prepared to deal with the misdiagnosis rate of epilepsy, which has been reported to be as high as 23% [30]. Understanding the differential diagnoses and the role of advanced diagnostic techniques, such as EEG and neuroimaging, is essential in ensuring correct diagnoses and effective management. The educational value of this review lies in its ability to equip future clinicians with the knowledge necessary to navigate these complexities and improve patient care in both acute and long-term settings.

## Conclusions

In conclusion, mastering the complexities of epilepsy is vital for UK medical students in their future roles as clinicians. Epilepsy is not only a prevalent condition in the UK but also one that poses considerable diagnostic and therapeutic challenges. The high proportion of patients who do not respond to existing AEDs highlights the need for students to understand both current treatment protocols and the ongoing advancements in seizure management. Students must also recognise the importance of socioeconomic factors in the presentation and management of epilepsy, preparing them to address these disparities in their future clinical practice. This review underscores the necessity for UK medical students to stay informed on the latest developments in epilepsy research and treatment. With advances in diagnostic technologies, genetics, and personalised medicine on the horizon, future doctors will need to apply an evidence-based approach tailored to individual patient needs. By gaining a broad understanding of epilepsy's pathophysiology, classification, and management, UK medical students can become better equipped to meet the challenges posed by this complex neurological disorder in their professional careers.

Overall, this review covered the history, etymology, and definition of epilepsy, as well as the distinction between seizures and epilepsy. The causes of provoked seizures were discussed, along with the epidemiology of epilepsy and its impact on the UK economy. The classification of epilepsy was explored based on the ILAE 2017 guidelines. Additionally, the pathophysiology of epilepsy was discussed, with a focus on the well-established theory of excitatory and inhibitory mechanisms. Key aspects of the clinical features and diagnosis of epilepsy were addressed, emphasising the importance of obtaining a thorough history and conducting a physical examination, as well as the benefits of laboratory investigations, neuroimaging, and EEG. Finally, guided by the UK NICE guidelines, this review explored the management of epilepsy and acute seizures and briefly discussed non-pharmacological treatments for refractory epilepsy. The emerging potential of the ketogenic diet and vagus nerve stimulation as therapeutic options was also highlighted.
